# Economic evaluation of using polygenic risk score to guide risk screening and interventions for the prevention of type 2 diabetes in individuals with high overall baseline risk

**DOI:** 10.3389/fgene.2022.880799

**Published:** 2022-09-15

**Authors:** Janne Martikainen, Aku-Ville Lehtimäki, Kari Jalkanen, Piia Lavikainen, Teemu Paajanen, Heidi Marjonen, Kati Kristiansson, Jaana Lindström, Markus Perola

**Affiliations:** ^1^ School of Pharmacy, University of Eastern Finland, Kuopio, Finland; ^2^ Department of Public Health and Welfare, Finnish Institute for Health and Welfare, Helsinki, Finland; ^3^ Research Program for Clinical and Molecular Metabolism, Faculty of Medicine, University of Helsinki, Helsinki, Finland

**Keywords:** type 2 diabetes, prevention, polygenic risk score, cost-effectiveness, QALY

## Abstract

Type 2 diabetes (T2D) with increasing prevalence is a significant global public health challenge. Obesity, unhealthy diet, and low physical activity are one of the major determinants of the rise in T2D prevalence. In addition, family history and genetic risk of diabetes also play a role in the process of developing T2D. Therefore, solutions for the early identification of individuals at high risk for T2D for early targeted detection of T2D, prevention, and intervention are highly preferred. Recently, novel genomic-based polygenic risk scores (PRSs) have been suggested to improve the accuracy of risk prediction supporting the targeting of preventive interventions to those at highest risk for T2D. Therefore, the aim of the present study was to assess the cost-utility of an additional PRS testing information (as a part of overall risk assessment) followed by a lifestyle intervention and an additional medical therapy when estimated 10-year overall risk for T2D exceeded 20% among Finnish individuals screened as at the high-risk category (i.e., 10%–20% 10-year overall risk of T2D) based on traditional risk factors only. For a cost-utility analysis, an individual-level state-transition model with probabilistic sensitivity analysis was constructed. A 1-year cycle length and a lifetime time horizon were applied in the base-case. A 3% discount rate was used for costs and QALYs. Cost-effectiveness acceptability curve (CEAC) and estimates for the expected value of perfect information (EVPI) were calculated to assist decision makers. The use of the targeted PRS strategy reclassified 12.4 percentage points of individuals to be very high-risk individuals who would have been originally classified as high risk using the usual strategy only. Over a lifetime horizon, the targeted PRS was a dominant strategy (i.e., less costly, more effective). One-way and scenario sensitivity analyses showed that results remained dominant in almost all simulations. However, there is uncertainty, since the probability (EVPI) of cost-effectiveness at a WTP of 0€/QALY was 63.0% (243€) indicating the probability that the PRS strategy is a dominant option. In conclusion, the results demonstrated that the PRS provides moderate additional value in Finnish population in risk screening leading to potential cost savings and better quality of life when compared with the current screening methods for T2D risk.

## Introduction

Type 2 diabetes (T2D) is a significant global public health challenge. Currently, around 460 million persons are at risk of T2D and the total number of people living with diabetes is projected to rise to 643 million by 2030 (International Diabetes Federation, 2021). Population aging, overweight and obesity associated with excess energy intake, Western dietary habits, and low physical activity are the major determinants of the rise in T2D prevalence. In addition, family history of diabetes plays a role in the process of developing T2D, since the lifetime risk of T2D in people with one parent having T2D is 40% and up to 70% if both parents have it (Groop et al., 1996). Due to this adverse development, societal costs and health care expenditures are expected to grow significantly (Williams et al., 2020). Therefore, solutions for identification of individuals at high risk for T2D for targeted prevention interventions and early detection of T2D are highly needed.

Due to resource constraints, it is important to obtain reliable risk information on individuals’ risk of disease to be able to target preventive interventions in a cost-effective way. For example, the Finnish diabetes risk score (FINDRISC) is a widely used risk assessment tool estimating the 10-year overall risk of developing T2D based on traditional risk factors, such as age, body mass index, physical activity, and family history of diabetes (Lindström and Tuomilehto, 2003). Besides above well-known conventional risk factors, T2D has a strong genetic component (Wray et al., 2013; Khera et al., 2019; Pärna et al., 2020). Therefore, over the last decade, development of novel genomic-based polygenic risk scores (PRSs) has increased the potential of using genomic information in the risk screening and prevention of T2D (Padilla-Martínez et al., 2020). PRSs summarize hundreds of thousands of risk-increasing and risk-decreasing genetic variants, along with their magnitude of impact, into a single measure of disease susceptibility. Higher than average PRSs indicate increased genetic risk of disease compared to the average genetic risk of the population. However, regardless of the potential advantages of PRSs in risk prediction, current evidence about its clinical utility and—particularly—cost-effectiveness (based on the current price levels of genotyping chip arrays) guiding risk stratification and the targeted preventive interventions against T2D has remained limited.

Currently, the advantages of the FINDRISC are its eligibility, easy access, and inexpensive way to screen an individual’s T2D risk based on the traditional risk factors. However, the use of PRS in addition to traditional T2D risk screening of a person gives an opportunity to increase the accuracy of estimating the future risk of T2D. This, in turn, may support more efficient allocation of limited health care resources by targeting lifestyle interventions and additional medical therapies for those at the highest risk (i.e., ≥ 20% 10-year risk of T2D). Therefore, the aim of the present study was to assess the cost-utility (as compared to an usual practice based on screening of traditional risk factors) of an additional PRS testing followed by a lifestyle intervention and an additional medical therapy when estimated 10-year overall risk for T2D exceeded 20% (i.e., an assumed threshold value for receiving interventions) among individuals screened as at the high-risk category (i.e., 10%–20% 10-year overall risk of T2D) based on traditional risk factors.

## Materials and methods

### Model overview

**FIGURE 1:**
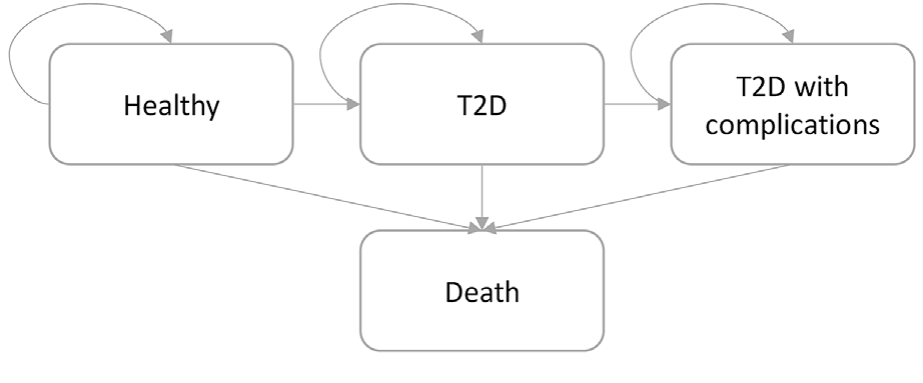
State-transition structure of an individual-level state-transition model. Simulated individuals transit in the model following the arrow direction. Simulation is concluded when all simulated individuals have transit to the “Death” state or when 100 years-of-age is reached, whichever comes first.

To estimate the cost-utility of the targeted PRS-based strategy when compared to the usual strategy in Finnish adult population, an individual-level state-transition model was constructed. The developed model included four mutually exclusive health states (i.e., Healthy, T2D, T2D with complications, and Death). Possible state-transitions between the modelled health states are illustrated in Figure 1. In the model, individuals developing T2D could develop further T2D-related complications, they might die, or they might survive to the next year in the T2D state (i.e., 1-year cycle length was applied in the model) without any event. In the present study, the individual-level modelling approach was applied to overcome the Markov assumption (Briggs et al., 2006), which relates to the fact that future transitions between health states are not dependent on previous states. Therefore, the individual-level state-transition modelling was applied to incorporate the memory of events occurring for simulated individuals in the model. Finally, the developed model was used to estimate the expected number of new T2D cases and associated consequences (in terms of costs and QALYs) with and without using PRS-based information to improve targeting of lifestyle and medical intervention using a lifetime time horizon.

In the developed model, individuals were passed through the model one-by-one, their results were stored and then the expected values of a cohort were obtained by aggregating the individual results. To reflect adequately both stochastic variability (i.e., individual-level variation) and parameter uncertainty (Degeling et al., 2017), in total 1,000 independent cohorts with 20,000 individuals each (i.e., 10,000 individuals per study arm) were simulated to obtain estimates for a cost-utility analysis. The model was written in R (R Core Team, 2021). See Supplementary Material S1 for further technical details.

### Target population

In the base-case scenario, the model was populated based on the real-world characteristics of the Finnish population aged 30–79 years without T2D at baseline (*n* = 2.97 million Finnish adults in 2017) but having high overall risk (i.e., 10%–20%) for developing T2D during the next 10 years measured with the FINDRISC. Based on these criteria, a total of 10.5% (*n* = 313,000) of the Finnish adult population was estimated to belong to the target population of the present study. At baseline, the average age of this target population was 61.5 years and 63.9% were men. The baseline distribution of FINDRISC in the modeled target population is shown in Supplementary Material S2. In the present study, the FINDRISC score was divided in five age- and sex-specific categories (i.e., from low risk to very high risk) indicating the 10-year risk of T2D.

### Estimating the baseline risk of type 2 diabetes with and without polygenic risk score

The baseline risk of T2D was estimated based on dataset obtained from The National FINRISK Study (*n* = 15,868), which was further enriched with a 10-year data from the national medicine reimbursement registry maintained by the Social Insurance Institution of Finland. From this register-based dataset, all new reimbursement rights and/or the ﬁrst purchases for T2D medicines were recorded and used as an indicator for a T2D diagnosis in the final analysis dataset. Finally, a parametric survival regression modelling was applied to estimate the 10-year risk of T2D based on baseline age, sex, and FINDRISC, which in turn was estimated based on age, body mass index (kg/m2), use of blood pressure medication, history of high blood glucose, physical activity, daily consumption of vegetables, fruits, or berries, as well as family history of diabetes (Lindström and Tuomilehto, 2003). Among the applied parametric regression models, the Weibull survival regression model was considered to provide the most reliable fit according to the AIC and BIC criteria, as well as visual inspections of the estimated survival curves. In addition, similar statistical analyses were also conducted using PRS as an additional predictor (i.e., together with age, sex, and FINDRISC) to be able to assess the baseline risk of T2D with and without genetic risk information. In these analyses we used a previously published PRS, calculated with LDPred algorithm and validated and tested in the UK Biobank population (120,280 and 288,978 participants, respectively) (Khera et al., 2018). In the FINRISK dataset, the PRS was then summarized from 6.9 million genetic variants. For the statistical analyses, the PRS was standardized to a normal distribution with mean of 0 and standard deviation of 1. The PRS approach is described in more detail in a recently published study (Marjonen et al., 2021).

The use of parametric survival models enabled the extrapolation of event risks over the actual 10-year follow-up period and thus, annual age- and sex-speciﬁc transition probabilities applied in the developed individual-level state-transition model were estimated based on these estimated and extrapolated incidence rates. The coefﬁcients of the Weibull regressions (with and without PRS) for the incidence of T2D are shown in Supplementary Material S3.

### Estimating the risk of type 2 diabetes with complications

To model the risk of T2D-related micro- and macrovascular complications in persons with newly diagnosed T2D, a previously developed Weibull survival regression model was applied (Martikainen et al., 2021). The coefﬁcients of the Weibull regression for the incidence of T2D-related complications is shown in Supplementary Material S4.

### Risk of death

To model the risk of overall mortality, the national all-cause life tables for men and women were used to characterize the risk of death conditional on age and sex. In addition, the risk of death in the modeled “T2D” and “T2D with complications” health states were adjusted to consider the increased risk of death in those health states by applying previously published HRs (Taylor et al., 2013; Weir et al., 2016).

### Interventions

In the present study, individuals with a screened ≥ 20% 10-year overall risk for T2D (i.e., very high risk of T2D) were assumed to have been directed to participate a lifestyle intervention and an additional medical therapy, if eligible with the national reimbursement criteria for medicines used along with diet and exercise to help manage weight in adults.

The hypothetical lifestyle intervention was assumed to consist of diet changes and increase in physical activity leading to a modest (i.e., from 2.5% to 4.9%) weight loss over a period of 1 year (Dunkley et al., 2014; Cameron Sepah et al., 2017; Castro Sweet et al., 2018) and its effect was assumed to last for 15 years (Lindström et al., 2013). Weight loss has been shown to be significantly associated with a reduced risk of T2D (Li et al., 2008; Lindström et al., 2013). Therefore, the effectiveness of the lifestyle intervention used in the model was conveyed through weight loss in kg.

In Finland, a glucagon-like peptide-1 receptor agonist, liraglutide 3 mg daily in combination with diet and exercise is currently reimbursed for individuals with BMI ≥ 35 kg/m^2^ and impaired glucose tolerance (IGT), as well as with medication treated hypertension or dyslipidemia. In addition, a naltrexone–bupropion combination therapy is also currently reimbursed for the treatment of obesity for individuals with BMI ≥ 40 kg/m^2^ or BMI ≥ 35 kg/m^2^ and with medication for T2D, dyslipidemia, or hypertension. Therefore, for simplicity, it was assumed that 30% of persons receiving a lifestyle intervention would also be applicable to receive a medical intervention which was estimated to result in a weight loss of over 5% (Lundgren et al., 2021). This proportion of individuals receiving a medical intervention was estimated based on The FinHealth 2017 Study results, where over 30% of persons aged over 55 and FINDRISC over 15 (i.e., high risk of T2D) had hyperglycemia (Koponen et al., 2018). Finally, the association between the weight loss and the reduction in the risk of T2D was estimated based on a post-hoc analysis of Finnish DPS study data which is described in detail in a previous study (Jalkanen et al., 2021). The estimated average risk reduction applied in the model was 26% (HR = 0.74). The clinical parameters used in the model are shown in Table 1.

**TABLE 1:** Clinical parameters applied in the model, their distributions and the values used to estimate the distributions.

Parameter	Value (variation)^a^	Distribution	Distribution values used in PSA mean (SE)	Source
Effect of lifestyle intervention HR (95% CI)	0.74 (0.53–1.03)	Lognormal	0.74 (0.17)	Estimated based on the obtained weight loss
Effect of medical and lifestyle intervention HR (95% CI)	0.51 (0.37–0.69)	Lognormal	0.51 (0.10)	Estimated based on the obtained weight loss
T2D-specific mortality risk HR (95% CI)	Women: 2.47 (2.23–2.72) Men: 1.93 (1.79–2.07)	Lognormal	2.47 (0.12) 1.93 (0.07)	Taylor et al. (2013)
Mortality risk associated with T2D with complications	2.36 (1.70–3.29)	Lognormal	2.36 (0.34)	Weir et al. (2016)
All-cause mortality	Based on age and sex	—	—	Statistics Finland (2021)

a For variables without available confidence interval, a variation of ± 25% has been used as an estimate. In these cases, SE has been calculated as: SE = (upper bound−lower bound)/(1.96 × 2). PSA, probabilistic sensitivity analysis.

### Resource utilization and cost estimates

In the present study, the societal perspective (excluding direct non-medical costs, such as travel costs associated with the utilization of health care services) was applied in the study. The excess primary health care cost estimates of T2D were obtained from a recent Finnish study (Martikainen et al., 2021). The excess secondary health care costs of T2D, its complications and T2D-related productivity losses for persons under the age of 65 were based on previous Finnish studies (Koski et al., 2017; Koski et al., 2018a; Koski et al., 2018b). In addition, the annual average (per-person) costs of liraglutide treatment (used as a proxy for all medical therapies) and T2D medication (ATC-code A10) costs were obtained from the national medicine statistics maintained by the Social Insurance Institution of Finland. The intervention costs of the lifestyle counselling intervention was based on a Finnish lifestyle coaching program for obesity (Väätäinen et al., 2019). Since the PRS testing is not generally available in the Finnish public health care, in the base case analysis it was assumed that the unit cost of a PRS test is 50 Euro per test. Finally, all costs were adjusted to the 2021 price level using the official health care price index determined by Statistics Finland. All applied cost estimates are presented in Table 2.

**TABLE 2:** Cost estimates and their distributions applied in the model.

Cost parameter	Value (€) (variation)	Distribution applied in PSA	Distribution values used in PSA (€) mean (SE)	Source
Costs from productivity losses due to T2D^a^	7632 (5724–9540)	Gamma	7632 (974)	Koski et al. (2018b)
Cost of T2D complications^b^	4401 (3301–5501)	Gamma	4401 (561)	Koski et al. (2018a)
Additional health care costs of T2D excluding primary health care^b^	3315 (2486–4144)	Gamma	3315 (423)	Koski et al. (2017)
Cost of a medical therapy (annual)	1965	—	1965	Kela (2021)
Cost of intervention^b^	650 (488–813)	Gamma	650 (83)	Väätäinen et al. (2019)
Cost of polygenic risk score test	50	—	50	Assumption
Additional T2D health care costs for basic health care	562 (SD 575) for men 542 (SD 635) for women	Gamma	Men = 562 (9.53) women = 542 (9.82)	Martikainen et al. (2021)
Additional medication costs of T2D^b^	584 (438–730)	Gamma	584 (74)	Kela (2020)

a For persons under 65 years old.

b For variables without available confidence interval, a variation of ± 25% has been used as an estimate. In these cases, SE was calculated as: SE = (upper bound−lower bound)/(1.96 × 2). PSA, probabilistic sensitivity analysis.

### Utility estimates

Utility estimates applied to estimate the number of quality-adjusted life years (QALYs) achieved in the target population were obtained from previously published studies (Table 3). Briefly, the published population-level EQ-5D-3L utility values (stratified by age and sex) were applied to represent the average health-related quality of life in the target population (Saarni et al., 2006; Martikainen et al., 2011). Furthermore, EQ-5D-3L-based disutility weights (Clarke et al., 2002; Bagust and Beale, 2005; Solli et al., 2010; Kontodimopoulos and Pappa, 2012; Beaudet et al., 2014) were applied to adjust the impacts of T2D and its complications on the health-related quality of life. These disutility values were estimated as a weighted average, where disutility values associated with a single complication were weighted by their observed incidences between years 2000 and 2017 in Finland (Arffman et al., 2020).

**TABLE 3:** Utility parameters applied in the Markov model, their distributions and the values used to estimate the distributions.

Utilities	Value (variation)^a^	Distribution	Distribution values used in PSA mean (SE)		Source
Baseline utilities (EQ-5D-3L)	Women (age, utility, SE) 30–44: 0.906 (0.003) 45–54: 0.865 (0.005) 55–64: 0.810 (0.006) 65+: 0.770 (0.008) men (age, utility, SE) 30–44: 0.917 (0.003) 45–54: 0.876 (0.005) 55–64: 0.821 (0.006) 65+: 0.781 (0.008)	Beta		Alpha term (age, value) women 30–44: 8573 45–54: 4040 55–64: 3463 65+: 2130 men 30–44: 7755 45–54: 3806 55–64: 3351 65+: 2087	Beta term (age, value) women 30–44: 889 45–54: 631 55–64: 812 65+: 636 men 30–44: 702 45–54: 539 55–64: 731 65+: 585		Martikainen et al. (2011)
Disutility of T2D (EQ-5D-3L) (SE)	0.041 (0.012)	Beta		Alpha term 11.15	Beta term 260.9		Saarni et al. (2006)
Weighted disutility of T2D complications (EQ-5D-3L)	0.119 (0.078–0.160)	Beta		Alpha term 55.45 Beta term 410.50			Disutility values of individual complications (Clarke et al., 2002; Bagust and Beale, 2005; Solli et al., 2010; Kontodimopoulos and Pappa, 2012; Beaudet et al., 2014) proportion of complications (Arffman et al., 2020)

a For variables without available confidence interval, a variation of ± 25% has been used as an estimate. In these cases, SE has been calculated as: SE = (upper bound−lower bound)/(1.96 × 2). PSA, probabilistic sensitivity analysis.

### Base-case analysis

In the present study, QALY was considered as a primary effectiveness outcome. Expected average costs and QALYs were estimated for both interventions. In the cost-utility analysis, it was first checked if the targeted PRS-based strategy is a dominant option (i.e., having higher QALYs at a lower cost vs. the usual strategy). In case of non-dominance, an incremental cost-effectiveness ratio (ICER) was estimated.

In the base case, a life-time horizon, and a 3% discount rate per year for costs and QALYs (from the second year onward) were applied in accordance with the national HTA guidelines (Pharmaceuticals Pricing Board, 2019).

### Sensitivity analyses

To study the robustness of the obtained results, different one-way and scenario sensitivity analyses were conducted to assess the impact of changes in the input parameters on outcomes:

1) Effect of applied discount rate on ICER was studied by varying discount rates between 0% and 5%
2) Impact of applied time horizon on ICER was studied by using shorter time horizons (i.e., 10- and 20-years)
3) Effect of exclusion of productivity costs on ICER was studied by running the model only with direct health care costs.

In addition, to test the association between the assumed price of the PRS testing and the ICER, a previous calculation framework (Standaert et al., 2014) was applied to indicate at what price the ICER equals zero (i.e., no difference in total cost between interventions). This point was referred as the cost neutral point. Finally, as mentioned above, also a probabilistic sensitivity analysis (PSA) was applied to evaluate the impact of simultaneous variation in model parameters on the model results. The results of the PSA were presented using a cost-effectiveness acceptability curve (CEAC) calculated from the net monetary benefit statistic across a range of willingness-to-pay (WTP) thresholds (Fenwick et al., 2006). The CEAC describes the probability that the “true” ICER estimate will be below the selected WTP. Furthermore, to study the total amount that the decision-makers should be willing to pay to eliminate all uncertainty in the decision, the expected value of perfect information (EVPI) was estimated as a function of WTP values (Wilson, 2015).

## Results

### Base-case results

The expected costs and QALYs of all interventions are presented in Table 4. In the base-case, the use of the targeted PRS strategy improved the reclassification of individuals to the very-high risk category (i.e., a risk category receiving a lifestyle intervention and an additional medical therapy in the present study), on average, by 12.4 percentage points among individuals who would otherwise not been included in these activities using the usual practice. This, in turn, increased the number of years without T2D and T2D-related complications, on average, by 0.72% and 2.7%, respectively. In addition, in the long-term period, the use of the targeted PRS strategy decreased the risk of overall death by 2.0%, on average, as compared to the usual practice. These above changes resulted in cost savings, on average, −253€ per person as compared to the usual practice. In addition, the targeted PRS strategy produced, on average, 0.022 additional QALYs per person as compared to the usual practice. Thus, in the base-case, the targeted PRS-based strategy was a dominant intervention option with lower expected costs and a higher number of expected QALYs.

**TABLE 4:** Base-case results.

Strategy	Costs (€)	QALYs	Incremental costs (€)	Incremental QALYs	ICER
The usual practice	13619	12.13			
The targeted PRS strategy	13373	12.15	−253	0.022	Dominant option^a^

a The targeted PRS strategy is less costly, and more effective.

### One-way sensitivity and scenario analyses

The results of one-way sensitivity analyses showed that the largest effect on the cost-utility results was the discount rate that was used with 0% discount resulting in cost savings of 444€ and 0.038 QALYs, whereas the use of 5% discount rate resulted savings of 165€ and 0.016 QALYs. Based on the results of the base-case analysis, the cost neutral point (i.e., ICER-value 0 €/QALY) was reached with a PRS test price of 303€. When shorter time horizons (i.e., 10- and 20-year time horizons) were applied the corresponding points were 19€ and 273€. The results for sensitivity analysis are shown in Table 5.

**TABLE 5:** Results from sensitivity analyses.

Scenario	Incremental cost (€)	Incremental QALYs	ICER (€/QALY)	Cost neutral point (price of PRS test, €)
Discount 0%	−444	0.038	Dominant option^a^	494
Discount 5%	−165	0.016	Dominant option^a^	215
No productivity costs	−200	0.022	Dominant option^a^	250
10-year time horizon	31	0.003	10333	19
20-year time horizon	−223	0.014	Dominant option^a^	273

a The targeted PRS strategy is less costly, and more effective.

### Probabilistic sensitivity analysis and expected value of perfect information

**FIGURE 2:**
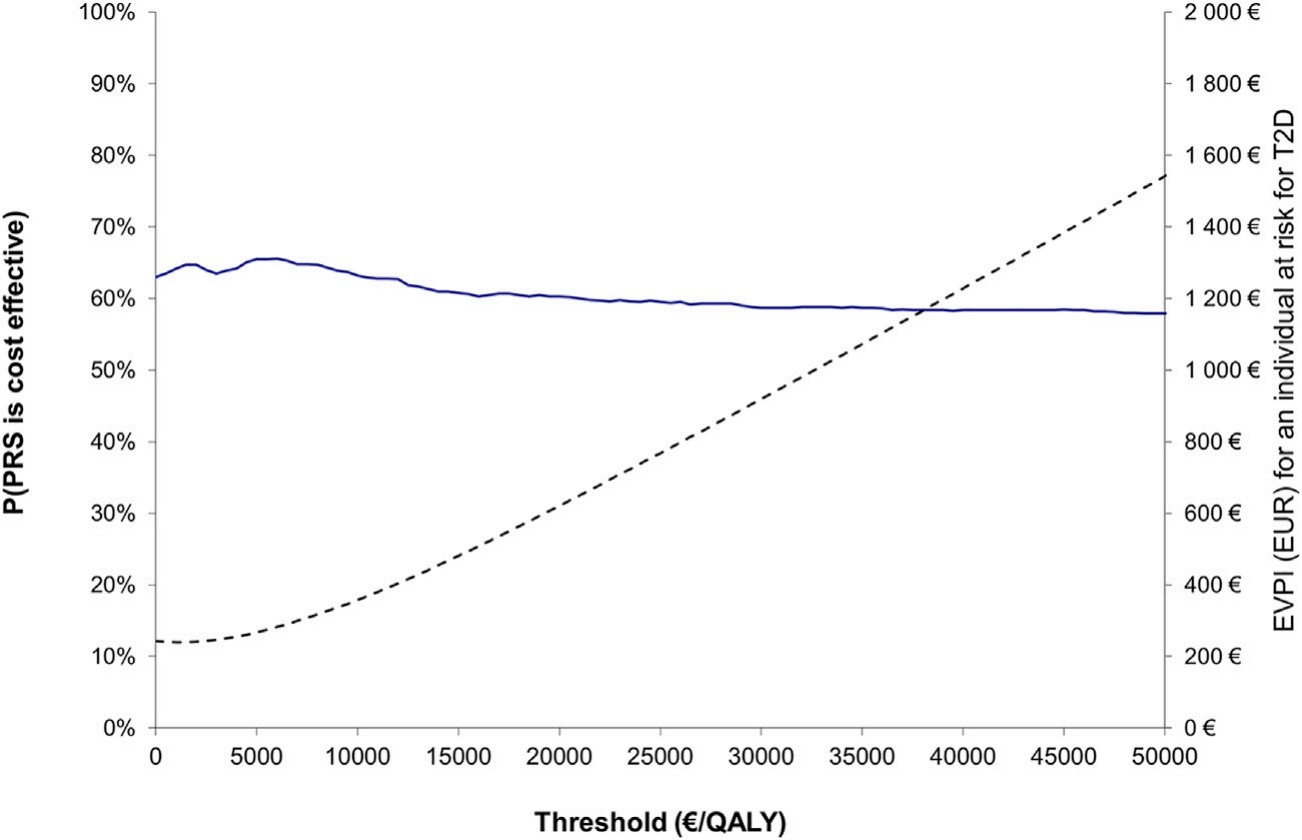
Cost-effectiveness acceptability curve (blue line; left y-axis) showing the probability that the PRS strategy is cost-effective compared to the usual practice, together with expected value of perfect information (dotted line; right y-axis) over a range of values for WTP.

The probability of the cost-effectiveness of the PRS strategy remained stable as the WTP threshold increased (Figure 2). The probability of cost-effectiveness at a WTP of 0€/QALY was 63.0% indicating the probability that the PRS strategy is a dominant option (i.e., less costly, and more effective) conditional on the parameter uncertainty of the applied model. The corresponding EVPI estimate at a WTP of 0€/QALY was 243€ indicating the expected cost of uncertainty and the expected opportunity loss that could be avoided with perfect information.

## Discussion

In the present study, the targeted PRS-based screening strategy reclassified around 12 percentage points of individuals to be very high-risk individuals who would have been originally classified as high-risk individuals using the usual practice only. This finding is in line with previous studies, where the additional information given by the PRS test alone has been significant but not as good when used in addition to traditional risk factors (Mars et al., 2020). The above reclassification estimate stands for around 38,000 persons of the 313,000 persons estimated to be at very high-risk in Finland. Our results show that performing PRS tests in this target population is a dominant strategy (i.e., a strategy associated with lower costs and better health outcomes), and this dominance could be expected to improve as the price of genetic testing keeps decreasing in the future. However, the clinical relevance of the expected proportion of reclassified individuals (when compared to the usual practice) and uncertainty associated with the cost-effectiveness estimates require further considerations. In the present study, the expected value of perfect information (at a WTP of 0€/QALY) in a comparison of the targeted PRS-based strategy versus the usual practice was around 243€ per an individual at high risk for T2D. This estimate could be considered as the theoretical value of additional research (per an individual at high risk for T2D) in eliminating all decision uncertainty related to the adaptation of the targeted PRS screening strategy in a case of individuals with high baseline risk for T2D, when the price of PRS testing is assumed to be 50 Euros per a test.

One of the strengths of our study is that we applied nationally representative data from the genetic background of the Finnish population to estimate the PRS score and its relationship with the incidence of T2D in the target population as an additional risk factor. To study this relationship, we applied parametric survival models, with the power of extrapolate to longer times outside the observed data, which supported our aims to project long-term economic and health outcomes. Currently, parametric survival regression models are the most common approach to extrapolate event risks over the actual follow-up time in the health economic modeling (Bell Gorrod et al., 2019; Gallacher et al., 2019). Furthermore, we also applied nationally representative estimates for T2D-related additional health care costs and productivity losses associated with T2D and its complications. Conducted deterministic scenario analyses showed that the inclusion of T2D-related productivity losses had a significant impact on the obtained cost-effectiveness results. This finding is in line with previous studies highlighting the significant role of productivity losses in T2D-related societal costs (Andersson et al., 2020; Kurkela et al., 2021).

There are also several limitations that need to be considered when interpreting the results of the present study. First, we defined threshold values for high (i.e., 10%–20% risk of T2D) and very high (>20% risk of T2D) risks of developing T2D within 10 years similarly (but using different risk factors) as defined in a recent Finnish study (Marjonen et al., 2021) due to the inclusion of PRS as an additional risk factor. These definitions separate from the original risk categories of the FINDRISC, which should be considered when interpreting the results. Second, we focused on to study the cost-utility of the targeted (two-stage) PRS-based strategy in individuals with high overall baseline risk for T2D based on the FINDRISC risk test and a hypothetical lifestyle counselling intervention supported by medical therapies. The results of the present study, however, are sensitive to changes in the baseline risk of T2D. Therefore, the PRS-based risk screening without the FINDRISC pre-screening would probably lead to less favorable cost-effectiveness results and higher budget impacts (i.e., because of a higher number of required PRS tests) at the population level due to lower baseline risk of T2D in the target population. Also due to the lack of transferability of PRS between different ethnic groups, the results of our study may be limited to Finnish population. Third, there is a lack of studies investigating the effect of the genetic background of the participants on the effect of the prevention of T2D. Thus, in the present study, we assumed that the effects of interventions were independent from the genetic risk of individuals. Fourth, lifestyle counselling interventions are not systematically available in the Finnish health care system currently and therefore, there might be limited possibilities to provide such services even for those individuals at highest risk in practice. However, digital solutions might provide a scalable solution for this challenge in the near future (Leväsluoto et al., 2021). In addition, for simplicity, we assumed full adherence to interventions, which might not be the case in the real-world situation among those individuals at highest risk (Dunkley et al., 2014), which in turn could impact on the effectiveness of interventions. However, the effect of adherence could be expected to be similar in both study arms, since communicating genetic risks of disease have not been shown to have significant impact on risk-reducing health behavior (Hollands et al., 2016), which reduce its impact on cost-effectiveness estimates. Also, we did not model the adverse effects of liraglutide since the most common adverse effects of liraglutide 3.0 mg are nausea and diarrhea (as compared to placebo) (Pi-Sunyer et al., 2015) and the evaluation of impacts of the most common adverse effects (i.e., nausea, diarrhea) on QoL is challenging and available data is limited. Therefore, for example, recently published studies have not considered the impacts of adverse effects of liraglutide (Shah et al., 2018; Lee et al., 2020; Hu et al., 2022). Thus, for simplicity, we did not consider the impacts of adverse effects on QoL or costs. Finally, if PRSs will eventually be translated to clinic, they will probably not be utilized for one disease only, but several for each individual, which lessens the cost per disease but of course makes this kind of analyses even more complex.

Overall, cost-effectiveness of genetic testing is a question which will become more and more relevant in the future. For example, a previous study has shown that earlier genetic testing has not been considered to be cost-effective when screening for mature-onset diabetes of the young when the cost of a genetic test was $2,580 (Naylor et al., 2014). However, lately the price of genetic testing has decreased considerably with the introduction of biochips and different kinds of assays (Locke et al., 2020). Therefore, the adaptation of an iterative approach (Sculpher et al., 1997; Boyd et al., 2010; Buchanan et al., 2013) to economic evaluation where the cost-effectiveness estimates are updated regularly based on a systematic process of information gathering and the updated price levels of genetic testing could inform decision-making in a timely manner in the rapidly evolving field of genetic testing. However, the cost-effectiveness is just one decision criteria, since the implementation of genetic risk information in public health involves also other issues, such as approaches to communicate personalized risk information (Marjonen et al., 2021), as well as ethical and social psychological (Fulda and Lykens, 2006) considerations as a part of implementation processes.

As a summary, the findings from this economic evaluation study suggest that the targeted two-stage polygenic risk screening in individuals with high overall baseline risk of T2D could improve the detection of individuals with very high risk of T2D, which in turn leads to lower expected costs and a higher number of QALYs when compared to the usual screening strategy based on the traditional risk factors in the Finnish health care system.

## Data Availability

The datasets presented in this article are not readily available because the data that support the findings of this study are available from the Finnish Institute for Health and Welfare (THL), but restrictions apply to the availability of these data, which were used under license for the current study, and so are not publicly available. Data are however available from the authors upon reasonable request and with permission of THL. Requests to access the datasets should be directed to janne.martikainen@uef.fi.
