# Supplementary material for: Economic evaluation of using polygenic risk score to guide risk screening and interventions for the prevention of type 2 diabetes in individuals with high overall baseline risk
Source: Front Genet. 2022 Sep 15;13:880799. doi: 10.3389/fgene.2022.880799 (PMC9520240; doi:10.3389/fgene.2022.880799)
Supplement: Supplementary file 1 [file Table1.DOCX]

**Supplementary File S1.**

**Short, technical description of the simulation model**

The model was written in R (R Core Team, 2021). The model consisted of three hierarchical, nested levels: The first level was for the simulation, the second one was for a cohort, and the third one was for an individual. An object-oriented programming paradigm was chosen, so these three levels could be described as distinct classes, and corresponding objects could be created during the simulation run. The library R6 (Chang, 2020) was chosen to model these classes. The specs of the system used in the modeling were as follows:

4x Dell C6420 (servers 1-4)

CPU: 2 x Intel Xeon Gold 6148 (40 Cores / 80 Threads)

Memory:

3 Nodes 376 GB

1 Nodes 768 GB

LOCAL DISK (/scratch): 300 GB SSD

2x Lenovo SR670 v2 (servers 5-6)

GPU: 4x NVIDIA A100 40 GB

CPU: Intel Xeon Gold 6326 (32 Cores / 64 Threads)

RAM: 512 GB

LOCAL DISK (/scratch): 1.6 TB NVME

*Before the Nested Class Structure is Created*

For the sake of memory efficiency and effectiveness, the sampled cohort parameters such as different costs and utilities were not created with the corresponding cohort, but before the simulation.

*The Simulation Level*

The simulation has only one loop for creating the number of cohorts needed.

*The Cohort Level*

The cohort related features could have been created with the cohort, but contrary to this intuitive solution, the cohort parameters were sampled before the simulation was even started. This decision was made since it was found more memory efficient and effective. The individual parameters created here, during each iteration of the individual generation loop, included one PRS value (from a standard normal distribution) and FINDRISC class membership (from a multinomial distribution). Furthermore, these sampled values were passed to the individual (following the PRS strategy) and its clones (following the Current strategy).

*The Individual Level*

Modeling the individual lie at the heart of every microsimulation model. The individual class can be roughly divided in two parts: Before and during the lifetime simulation. Before the individual starts go through the annual cycles, some preparations take place. These include putting cohort parameters and individual parameters into more convenient form, but also performing virtually the FINDRISC test and the PRS test for the individual in question (**Figure 1**).


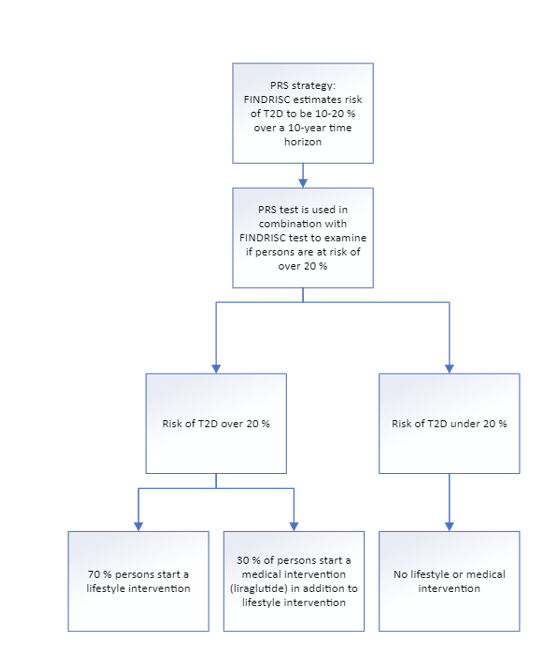


**Figure 1**. The structure of the model where PRS strategy and current strategy are compared on an individual level

The progression through life cycles was performed using a loop. On every iteration, costs, utilities, and current state is saved on a list. Finally, a corresponding row of a transition matrix, and this row was used a parameter vector for a multinomial random variable. The realization of the random variable would be the state where the individual would land on for the next iteration of the loop.

After the individual has finished its run, the results would be transferred back to the top through the levels of hierarchical structure: From the individual to the cohort, and from the cohort to the simulation. Since 10 000 individuals with their clones were sampled in each of the 1000 cohorts, the model would require a substantial amount of memory for the results in the raw form: One cell for every cost, utility and state of an individual and for every cycle. Additionally, it was reasoned and observed that the data frames contained many cells with either zero costs or zero utilities or the costs, utilities and states would repeat many times in a row within an individual. Therefore, a compression method was developed as follows, for example:

a*value1 | b*value2 | c*value3

This notation would be read as: First value1 is repeated a times, then value2 is repeated b times, and finally value3 is repeated c times. In other words, the asterisk character denotes repetition, and the vertical line serves a sequence separator.

The model was populated with a simple random sampling without replacement (srswor).

The correlation structure between the Weibull regression coefficients was also taken into consideration (**Table 1**), and the regression coefficients were assumed to be normally distributed.

**Table 1.** The correlations between the Weibull regression coefficients.

**Weibull regression coefficient correlations, risk of T2D according to FINDRISC:**

|  | **Constant term** | **Age coefficient** | **Sex coefficient** | **FINDRISC**  **7-11** | **FINDRISC 12-14** | **FINDRISC 15-19** | **FINDRISC 20+** | **Gamma** |
| --- | --- | --- | --- | --- | --- | --- | --- | --- |
| **Constant term** | 1.000 | -0.522 | -0.297 | -0.420 | -0.425 | -0.385 | -0.171 | 0.687 |
| **Age coefficient** | -0.522 | 1.000 | 0.000 | -0.220 | -0.219 | -0.257 | -0.041 | -0.093 |
| **Sex coefficient** | -0.297 | 0.000 | 1.000 | 0.098 | 0.137 | 0.155 | 0.104 | -0.223 |
| **FINDRISC**  **7-11** | -0.420 | -0.220 | 0.098 | 1.000 | 0.821 | 0.785 | 0.220 | -0.446 |
| **FINDRISC 12-14** | -0.425 | -0.219 | 0.137 | 0.821 | 1.000 | 0.766 | 0.222 | -0.506 |
| **FINDRISC 15-19** | -0.385 | -0.257 | 0.155 | 0.785 | 0.766 | 1.000 | 0.222 | -0.549 |
| **FINDRISC 20+** | -0.171 | -0.041 | 0.104 | 0.220 | 0.222 | 0.222 | 1.000 | -0.212 |
| **Gamma** | 0.687 | -0.093 | -0.223 | -0.446 | -0.506 | -0.549 | -0.212 | 1.000 |

**Weibull regression coefficient correlations, risk of T2D according to FINDRISC + PRS regression:**

|  | **Constant term** | **Age coefficient** | **Sex coefficient** | **FINDRISC**  **7-11** | **FINDRISC**  **12-14** | **FINDRISC**  **15-19** | **FINDRISC**  **20+** | **PRS**  **Coefficient** | **Gamma** |
| --- | --- | --- | --- | --- | --- | --- | --- | --- | --- |
| **Constant term** | 1.000 | -0.530 | -0.298 | -0.411 | -0.409 | -0.364 | -0.165 | -0.336 | 0.699 |
| **Age coefficient** | -0.530 | 1.000 | 0.000 | -0.223 | -0.224 | -0.262 | -0.035 | 0.113 | -0.093 |
| **Sex coefficient** | -0.298 | 0.000 | 1.000 | 0.097 | 0.130 | 0.140 | 0.103 | 0.087 | -0.225 |
| **FINDRISC**  **7-11** | -0.411 | -0.223 | 0.097 | 1.000 | 0.816 | 0.780 | 0.216 | 0.112 | -0.431 |
| **FINDRISC 12-14** | -0.409 | -0.224 | 0.130 | 0.816 | 1.000 | 0.759 | 0.215 | 0.098 | -0.482 |
| **FINDRISC 15-19** | -0.364 | -0.262 | 0.140 | 0.780 | 0.759 | 1.000 | 0.214 | 0.113 | -0.525 |
| **FINDRISC 20+** | -0.165 | -0.035 | 0.103 | 0.216 | 0.215 | 0.214 | 1.000 | 0.059 | -0.204 |
| **PRS Coefficient** | -0.336 | 0.113 | 0.087 | 0.112 | 0.098 | 0.113 | 0.059 | 1.000 | -0.359 |
| **Gamma** | 0.699 | -0.093 | -0.225 | -0.431 | -0.482 | -0.525 | -0.204 | -0.359 | 1.000 |

**Weibull regression coefficient correlations, rate of T2D complications:**

|  | **Age Coefficient** | **Sex Coefficient** | **Constant term** |
| --- | --- | --- | --- |
| **Age Coefficient** | 1 |  |  |
| **Sex Coefficient** | 0.284 | 1 |  |
| **Constant term** | -0.911 | -0.630 | 1 |
