# Supplementary material for: Economic evaluation of using polygenic risk score to guide risk screening and interventions for the prevention of type 2 diabetes in individuals with high overall baseline risk
Source: Front Genet. 2022 Sep 15;13:880799. doi: 10.3389/fgene.2022.880799 (PMC9520240; doi:10.3389/fgene.2022.880799)
Supplement: Supplementary file 2 [file Table2.DOCX]

**Supplementary File S2.**

The FINDRISC score distribution in population with 10-20% risk of developing T2D within 10 years according the the FINDRISC regression:

| **Age Group** | **Gender** | **FINDRISC 0-6** | **FINDRISC 7-11** | **FINDRISC 12-14** | **FINDRISC 15-19** | **FINDRISC 20+** |
| --- | --- | --- | --- | --- | --- | --- |
| **30-39** | **Women** | 0.000 | 0.000 | 0.161 | 0.839 | 0.000 |
| **40-49** | **Women** | 0.000 | 0.000 | 0.253 | 0.747 | 0.000 |
| **50-59** | **Women** | 0.000 | 0.000 | 0.606 | 0.394 | 0.000 |
| **60-69** | **Women** | 0.000 | 0.000 | 0.860 | 0.140 | 0.000 |
| **70-79** | **Women** | 0.000 | 0.000 | 0.902 | 0.098 | 0.000 |
| **30-39** | **Men** | 0.000 | 0.000 | 1.000 | 0.000 | 0.000 |
| **40-49** | **Men** | 0.000 | 0.198 | 0.802 | 0.000 | 0.000 |
| **50-59** | **Men** | 0.000 | 0.460 | 0.540 | 0.000 | 0.000 |
| **60-69** | **Men** | 0.000 | 0.718 | 0.282 | 0.000 | 0.000 |
| **70-79** | **Men** | 0.000 | 0.822 | 0.178 | 0.000 | 0.000 |

The FINDRISC score distribution in the general population:

| **Gender** | **Age** | **FINDRISC 0-6** | **FINDRISC**  **7–11** | **FINDRISC 12-14** | **FINDRISC 15-19** | **FINDRISC 20+** |
| --- | --- | --- | --- | --- | --- | --- |
| **Man** | **30–39** | 0.738 | 0.214 | 0.048 | 0 | 0 |
| **Man** | **40–49** | 0.545 | 0.33 | 0.098 | 0.028 | 0 |
| **Man** | **50–59** | 0.288 | 0.412 | 0.178 | 0.104 | 0.017 |
| **Man** | **60–69** | 0.195 | 0.47 | 0.177 | 0.135 | 0.022 |
| **Man** | **70–79** | 0.193 | 0.484 | 0.155 | 0.143 | 0.025 |
| **Woman** | **30–39** | 0.687 | 0.216 | 0.073 | 0.022 | 0.001 |
| **Woman** | **40–49** | 0.506 | 0.352 | 0.093 | 0.045 | 0.005 |
| **Woman** | **50–59** | 0.266 | 0.425 | 0.185 | 0.105 | 0.019 |
| **Woman** | **60–69** | 0.163 | 0.444 | 0.237 | 0.126 | 0.003 |
| **Woman** | **70–79** | 0.13 | 0.379 | 0.228 | 0.224 | 0.039 |
