# Supplementary material for: Economic evaluation of using polygenic risk score to guide risk screening and interventions for the prevention of type 2 diabetes in individuals with high overall baseline risk
Source: Front Genet. 2022 Sep 15;13:880799. doi: 10.3389/fgene.2022.880799 (PMC9520240; doi:10.3389/fgene.2022.880799)
Supplement: Supplementary file 3 [file Table3.DOCX]

**Supplementary File S3.**

Coefficients of the Weibull regression for incidence of T2D according to the FINDRISC regression:

| **Parameter** | **Value** | **p-value** | **Distribution** | **Distribution values used in PSA**  **Mean (SE)** |
| --- | --- | --- | --- | --- |
| **Weibull regression coefficients, risk of T2D (95 % CI)** |  |  |  |  |
| Weibull Gamma | 1.346  (1.279 to 1.413) | <0.001 | Normal | 1.346  (0.034) |
| Sex Coefficient | -0.376  (-0.482 to -2.700) | <0.001 | Normal | -0.376  (0.054) |
| Age Coefficient | -0.008  (-0.014 to -0.002) | <0.01 | Normal | -0.008  (0.003) |
| Constant | 6.152  (5.784 to 6.520) | <0.001 | Normal | 6.152  (0.188) |
| FINDRISC 0-6 | 0.000  (0.000 to 0.000) | Reference | Normal | 0.000  (0.000) |
| FINDRISC 7-11 | -1.330  (-1.528 to 1.132) | <0.001 | Normal | -1.330  (0.101) |
| FINDRISC 12-14 | -1.740  (-1.965 to -1.515) | <0.001 | Normal | -1.740  (0.115) |
| FINDRISC 15-19 | -2.240  (-2.505 to -1.975) | <0.001 | Normal | -2.240  (0.135) |
| FINDRISC 20+ | -3.584  (-4.654 to -2.514) | <0.001 | Normal | -3.584  (0.546) |

The FINDRISC score groups (0-6, 7-11, 12-14, 15-19 and 20-26) are beta coefficients of the regression.

Coefficients of the Weibull regression for incidence of T2D according to the FINDRISC+PRS regression:

| **Parameter** | **Value**  **(variation)** | **p-value** | **Distribution** | **Distribution values used in PSA**  **Mean (SE)** |
| --- | --- | --- | --- | --- |
| **Weibull regression coefficients, risk of T2D** **(95 % CI)** |  |  |  |  |
| Weibull Gamma | 1.352  (1.285 to 1.419) | <0.001 | Normal | 1.352  (0.034) |
| Sex Coefficient | -0.380  (-0.486 to -0.274) | <0.001 | Normal | -0.380  (0.054) |
| Age Coefficient | -0.011  (-0.017 to -0.005) | <0.001 | Normal | -0.011  (0.003) |
| Constant | 6.284  (5.910 to 6.658) | <0.001 | Normal | 6.284  (0.191) |
| PRS Coefficient | -0.306  (-0.361 to -0.251) | <0.001 | Normal | -0.306  (0.028) |
| FINDRISC 0-6 | 0.000  (0.000 to 0.000) | Reference | Normal | 0.000  (0.000) |
| FINDRISC 7-11 | -1.269  (-1.463 to -1.075) | <0.001 | Normal | -1.269  (0.099) |
| FINDRISC 12-14 | -1.626  (-1.847 to -1.405) | <0.001 | Normal | -1.626  (0.113) |
| FINDRISC 15-19 | -2.010  (-2.271 to -1.749) | <0.001 | Normal | -2.010  (0.133) |
| FINDRISC 20+ | -3.410  (-4.472 to -2.348) | <0.001 | Normal | -3.410  (0.542) |

The FINDRISC score groups (0-6, 7-11, 12-14, 15-19 and 20-26) are beta coefficients of the regression.
