# Supplementary material for: Economic evaluation of using polygenic risk score to guide risk screening and interventions for the prevention of type 2 diabetes in individuals with high overall baseline risk
Source: Front Genet. 2022 Sep 15;13:880799. doi: 10.3389/fgene.2022.880799 (PMC9520240; doi:10.3389/fgene.2022.880799)
Supplement: Supplementary file 4 [file Table4.DOCX]

**Supplementary File S4.**

Weibull regression coefficients for the incidence of T2D-related complications:

| **Parameter** | **Value (variation)** | **p-value** | **Distribution** | **Distribution values used in PSA**  **Mean (SE)** |
| --- | --- | --- | --- | --- |
| **Weibull regression coefficients, rate of T2D complications** **(95 % CI)** |  |  |  |  |
| Weibull Gamma | 0.857  (0.778 to 0.944) | >0.001 | Normal | 0.857  (0.084) |
| Age Coefficient | -0.062  (-0.075 to -0.049) | >0.001 | Normal | -0.062  (0.063) |
| Sex Coefficient | -0.760  (-1.355 to -0.485) | >0.001 | Normal | -0.760  (0.444) |
| Complication Coefficient | 8.430  (7.290 to 9.570) | >0.001 | Normal | 8.430  (1.166) |
